# Possibility of screening for mild cognitive impairment via an eye tracking-based cognitive scale

**DOI:** 10.3389/fragi.2025.1532550

**Published:** 2025-06-27

**Authors:** Naoki Kodama, Sou Takahashi, Masazumi Tsuji, Yuji Kawase, Satoshi Naruse, Katsuya Urakami

**Affiliations:** ^1^ Department of Radiological Technology, Faculty of Medical Technology, Niigata University of Health and Welfare, Niigata, Japan; ^2^ Tsuji Internal Medicine, Cardiology, and Dentistry Clinic, Tokyo, Japan; ^3^ Kawase Neurology Clinic, Sanjo, Japan; ^4^ Midori Hospital, Niigata, Japan; ^5^ Department of Dementia Prevention, School of Health Science, Faculty of Medicine, Tottori University, Yonago, Japan

**Keywords:** mild cognitive impairment, screening, eye tracking-based cognitive scale, Alzheimer’s disease, virtual reality

## Abstract

**Introduction:**

The Montreal Cognitive Assessment (MoCA) is widely used as a screening test for mild cognitive impairment (MCI). However, the MoCA takes approximately 15 min to administer and evaluate by skilled examiners, such as medical professionals. This study assessed whether an eye tracking-based cognitive scale using virtual reality (VR) was accurate and efficient to screen for MCI.

**Methods:**

This study included 143 patients. The Virtual Reality-Based Cognitive Function Examination (VR-E) was used with all participants to evaluate their memory, judgment, spatial cognition, calculation, and language function.

**Results:**

Significant differences were observed in all cognitive domains of memory, judgment, spatial cognition, calculation, and language function between the Alzheimer’s disease (AD), MCI, and older healthy control (HC) groups. The area under the curve value of the VR-E score for the HC and MCI groups was 0.857, and that for the AD and MCI groups was 0.870. The correlation coefficient between the MMSE and VR-E scores was 0.566 (p < 0.001), and that between the Japanese version of the MoCA (MoCA-J) and VR-E scores was 0.648 (p < 0.001), which indicated a moderate correlation in both comparisons.

**Conclusion:**

The VR-E had the same diagnostic performance results as the MoCA-J, thus the VR-E has potential for use in screening patients for MCI.

## 1 Introduction

Early diagnosis of mild cognitive impairment (MCI) and Alzheimer’s disease (AD) is important. AD is the most common form of dementia, and its frequency increases in aging populations. MCI is commonly referred to as a pre-stage of dementia (Petersen, 2011; Petersen et al., 1999). Although dementia is typically irreversible, MCI is considered a transitional stage with some potential for reversibility. MCI progresses to dementia in 10%–20% of the cases yearly, highlighting the reason people with MCI are the most suitable candidates for dementia prevention from an outcome perspective (Bruscoli and Lovestone, 2004; Urakami, 2022). To reduce the societal incidence of dementia, it is essential to detect MCI early in medical settings and to implement community-based screening among older adults.

The Montreal Cognitive Assessment (MoCA), or the MoCA Japanese version (MoCA-J), is widely used as a screening tool for MCI (Nasreddine et al., 2005; Nasreddine et al., 2005). However, the MoCA and MoCA-J take approximately 15 min to administer and evaluate, and a skilled examiner, such as a medical professional is required for administration. Furthermore, the evaluation of the results may differ depending on examiner skill. Therefore, there is a need to develop MCI screening tools that do not require an examiner and can be performed easily and quickly in the community.

Recently, cognitive testing methods focused on eye movements have been developed (Crutcher et al., 2009; Gills et al., 2021). Readman et al. (2021) reviewed studies that evaluated the differences between older individuals with AD and MCI and older healthy controls (HC) using eye tracking. They reported eye tracking during natural tasks (reading, realistic simulations, searching for still images) could distinguish between HC and those with AD, and eye tracking has potential to be used in diagnosis and monitoring. However, they also reported that few papers included participants with MCI. In addition, Wolf et al. (2023) reviewed studies on eye movement-based gaze parameters of older adults with MCI and concluded these parameters have the potential to improve conventional cognitive assessment screening and assist in detecting early-stage AD. Oyama et al. (2019) reported an eye-tracking-based dementia assessment tool and found that the cognitive scores correlate well with the Mini-Mental State Examination (MMSE) (r = 0.74); the AD assessment scale-cognitive subscale and Frontal Assessment Battery also have had correlations between cognitive scores (r = −0.64, r = 0.54). Tadokoro et al. (2021) also assessed cognitive function via eye tracking and reported that the total score was significantly correlated with the MMSE score (r = 0.57). These studies compared their results with the MMSE but not with the MoCA or MoCA-J. FOVE, Inc., developed an eye-tracking technology based on advanced image processing. They developed a rapid dementia screening test to assess cognitive function from multiple cognitive domains by combining virtual reality (VR) video stimulation and high-precision eye-tracking technology (Chernyak et al., 2021), called the Virtual Reality-Based Cognitive Function Examination (VR-E). The VR-E is designed to assess cognitive function in middle-aged and older individuals by automatically analyzing their eye movements during self-guided tasks. It comprises fifteen items that assess cognitive function across five domains: memory, judgment, spatial cognition, calculation, and language.

Mizukami et al. (2023) reported the VR-E scores could distinguish the difference between participants with Clinical Dementia Rating (CDR) scale scores (Hughes et al., 1982) of CDR0 (no dementia) and CDR0.5 (suspected dementia). Their follow-up study built on those findings and determined the effectiveness of each individual cognitive domain of the VR-E in distinguishing between CDR0 and CDR0.5, as well as between CDR0.5 and CDR1 (Mizukami et al., 2024). However, because the CDR relies on subjective clinician judgment and informant interviews to stage dementia severity instead of objective cognitive test scores, CDR 0.5 alone does not provide a sufficient basis for confirming the presence of MCI. In contrast, the present study employed a more rigorous diagnostic protocol based on the widely recognized criteria of Petersen et al. (1999), which defines MCI using a combination of factors: memory complaints, preserved general cognition and daily functioning, objective memory impairment relative to age, and absence of dementia. This approach provides a more clinically objective standard for identifying MCI, making it possible to evaluate the effectiveness of the VR-E as a screening tool for early-stage cognitive decline more accurately. Therefore, this study extended beyond previous work by testing the ability of the VR-E to differentiate between individuals clinically diagnosed with MCI and healthy older adults implementing stricter, internationally accepted criteria.

## 2 Materials and methods

The study participants comprised 152 patients who visited three medical institutions (Midori Hospital, Kawase Neurology Clinic, and the Tsuji Internal Medicine, Cardiology and Dentistry Clinic) with memory loss as their main complaint. Nine of these 152 patients who had difficulty performing calibration due to ptosis or strabismus were excluded; thus 143 patients (mean age 77.8 ± 9.0 years, 58 men and 86 women) were included in the study. Among the 143 participants, 37 had AD, 84 had MCI, and 22 were older HC.

The diagnosis of MCI was established in line with Petersen et al. (1999); patients were required to exhibit a memory complaint, intact general cognitive function, normal daily activities, impaired memory relative to age, and have absence of dementia. A dementia diagnosis was determined using the Diagnostic and Statistical Manual of Mental Disorders, Fourth Edition (American Psychiatric Association, 1994), and the National Institute of Neurological and Communicative Disorders-Alzheimer’s Disease and Related Disorders Association diagnostic criteria for probable AD (McKhann et al., 1984). Healthy older adults were defined as those without history of brain disease and without vision or hearing impairment. The diagnoses of MCI and AD were made by a dementia specialist. All participants were assessed via the MMSE, MoCA-J, and VR-E on the same day; 16 of the 37 participants with AD and two of the 84 participants with MCI could not complete the MoCA-J. The details are presented in Table 1. The MMSE and MoCA-J were administered by experienced medical professionals (nurses, speech therapists, and clinical laboratory technicians).

**TABLE 1:** Participant information

Information	HC	MCI	AD	H-value	p-value	Multiple comparisons	
						HC vs. MCI	MCI vs. AD
Participants	22	84	37				
Age in years	69.5 ± 10.3 (22)	76.8 ± 7.7 (84)	84.8 ± 4.9 (37)	45.53	<0.001	<0.001	<0.001
M/W	5/17	40/44	12/25		0.059		
MMSE	29.3 ± 0.8 (22)	26.2 ± 2.1 (84)	20.3 ± 3.4 (37)	87.45	<0.001	<0.001	<0.001
MoCA-J	25.6 ± 2.0 (22)	20.0 ± 3.4 (82)	14.6 ± 3.0 (21)	60.55	<0.001	<0.001	<0.001
VR-E Score	86.6 ± 12.0 (22)	63.2 ± 22.6 (84)	28.0 ± 21.4 (37)	63.97	<0.001	<0.001	<0.001

Data are presented as mean ± standard deviation. The number in () represents the number of people inspected. The χ-square test was performed for sex. The Kruskal–Wallis test was performed for age, and MMSE, MoCA-J, and VR-E scores. The Steel–Dwass test was used to compare groups when significant differences were found.

AD, Alzheimer’s disease; HC, older healthy control; M, men; MCI, mild cognitive impairment; MMSE, Mini-Mental State Examination; MoCA, Montreal Cognitive Assessment; VR-E, Virtual Reality-Based Cognitive Function Examination; W, women.

The doctor provided all participants with written and verbal explanations, and written informed consent was obtained from them. This study was approved by the Research Ethics Review Committee of Niigata University of Health and Welfare (approval number: 18934–221020). The study was conducted in accordance with the Declaration of Helsinki.

**FIGURE 1:**
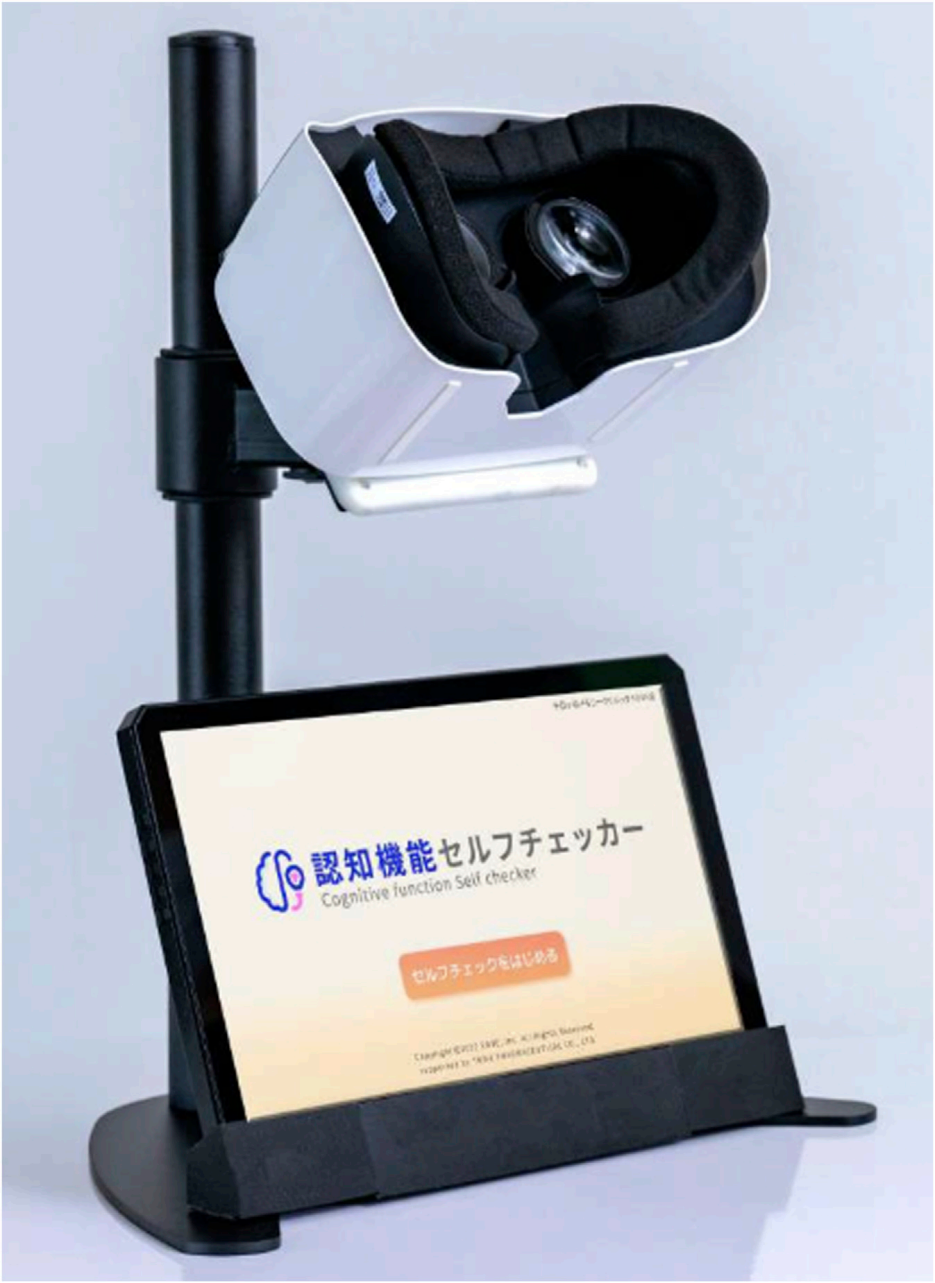
Appearance of the virtual reality-based cognitive function examination.

A photograph of the VR-E examination is shown in Figure 1. The examination started by having the participant look into a VR headset via built-in eye-tracking technology. Calibration was initially performed following the audio and text guidance instructions. Infrared light-emitting diodes (LED) with a wavelength of 850 nm placed around the eyepiece lens were used to irradiate the eyes. The reflected light was captured via a complementary metal oxide semiconductor sensor installed inside the unit, which captured and showed the participant’s eyes in the image projected onto the VR headset. The movement of the participant’s eyes was captured to track the coordinates of the participant’s viewpoint in the image projected onto the VR headset. During the calibration, the participant performed eye movements to follow a moving point on the VR screen to ensure high accuracy of the eye tracker during the assessment. After calibration, cognitive test questions and answer choices appeared before the visual field, and the questions and answers were explained via audio and text guidance. When the participant looked at the options they believed were the correct answer, their gaze movements within the VR screen were recorded, and on the basis of these recorded data, the participant’s cognitive function was calculated quantitatively by incorporating multiple numerical values, such as the time spent gazing at the correct option and the time taken to reach the correct option, into a specific function. The VR headset is a device whose power is shared with the personal computer through a USB port. It was certified by the American standard UL60950-1 and European standard IEC60950-1 for the safety of information technology equipment with a rated voltage of ≤600 V. In addition, because this equipment irradiates infrared LED light into the eyes, it was also certified according to EN 62471:2008, which is a safety assessment standard for the skin and eyes.

**FIGURE 2:**
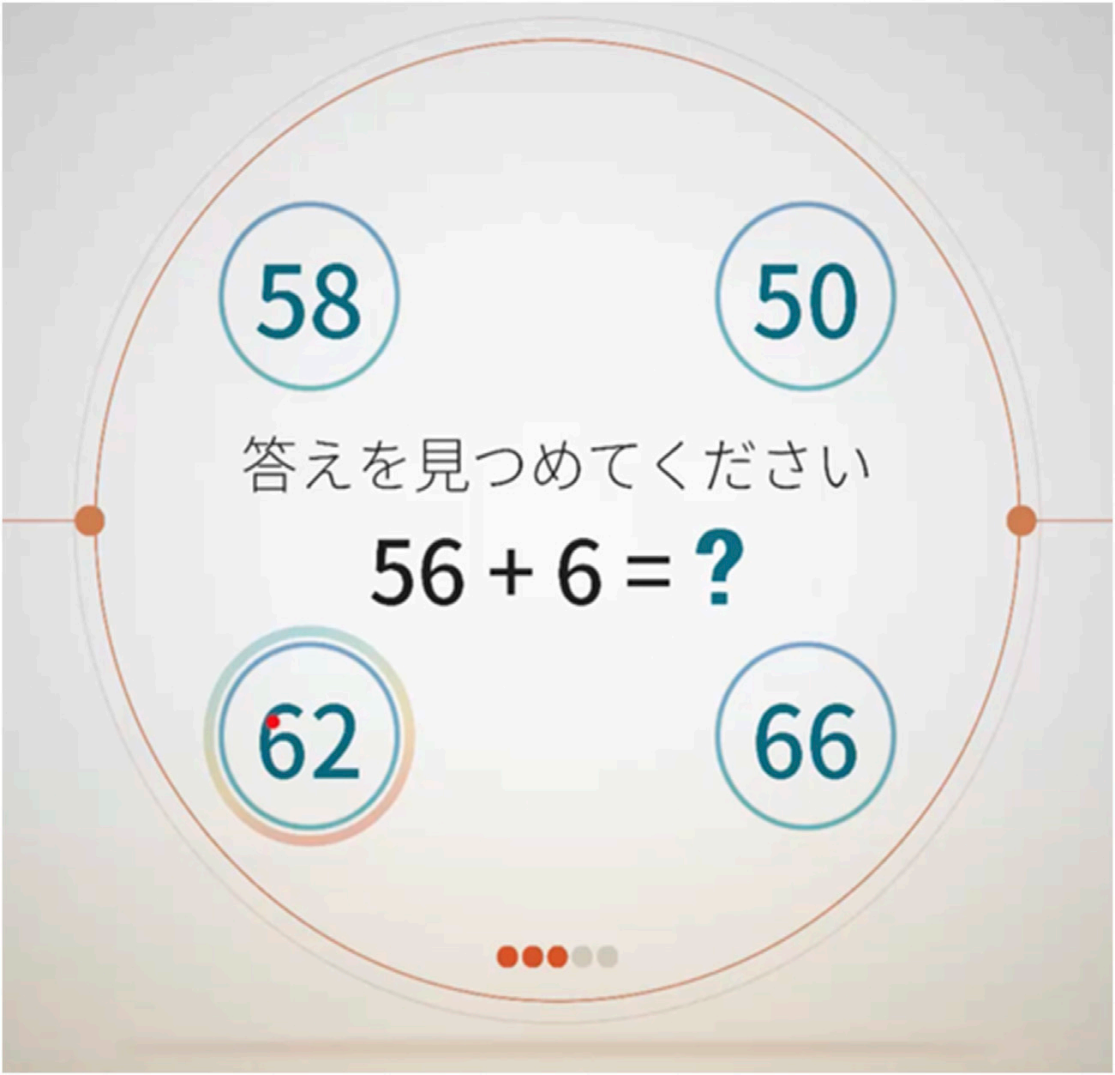
Virtual Reality-Based Cognitive Function Examination calculation task example. The instructions and questions are shown in the center with the four answer options arranged in circles surrounding the question.

The cognitive domains measured with the VR-E were memory, judgment, spatial cognition, calculation, and language function. In each memory task (3 in total), the participants memorized a specific pattern, and approximately 3 min later, they selected the pattern they had memorized from among multiple patterns. In each judgment task (4 in total), the participants selected the option that either belonged or did not belong in the presented group. The spatial cognition task (3 in total) involved two questions about guessing the number of blocks piled up and one about selecting a clock that shows a specified time. The calculation task (3 in total) had two questions that comprised four simple arithmetic operations and one to sum coin values (Figure 2). The language function task (2 in total) assessed comprehension of a given sentence.

The VR-E scores and VR-E cognitive domain scores were calculated based on 15 items in two steps. First, a score for each item was calculated mainly based on the proportion of gaze time spent on the correct answer. Second, the VR-E score was derived by averaging the values of all items, whereas the VR-E cognitive domain score was derived by averaging the values of items within each specific cognitive domain (memory, judgment, spatial cognition, calculation, and language function). All the scores are in the range of 0–100.

IBM SPSS Statistics 27.0 (IBM Corp., Armonk, NY, United States) was used for the statistical analyses. For the AD, MCI, and HC groups, the χ-square test was performed for sex, and Kruskal–Wallis test was performed for age, MMSE, MoCA-J, and VR-E scores. Steel–Dwass test was used to compare groups when significant differences were found. Receiver operating characteristic curves (ROCs) for the MoCA-J and VR-E scores were generated for the AD and MCI groups and for the MCI and HC groups, and the areas under the curve (AUCs) were determined. Spearman’s rank correlation coefficient for the MMSE, MoCA-J and VR-E scores were calculated. The significance level was set at p < 0.05.

## 3 Results

Among the AD, MCI, and HC groups, there was no significant difference in sex, although there was a significant difference in age (H = 45.53, p < 0.001); 69.5 ± 10.3, 76.8 ± 7.7, and 84.8 ± 4.9 years for the HC, AD, and HC groups, respectively. Significant differences were also observed between the MMSE (H = 87.49, p < 0.001), MoCA-J (H = 60.55, p < 0.001), and VR-E scores (H = 63.96, p < 0.001) among the three groups, suggesting consistent findings of distinct variations in cognitive function across these groups. The scores (0–100) of the five lower cognitive domains from the VR-E are listed in Table 2. Table 2 presents the significant differences between the three groups in all the specific cognitive domains of memory (H = 69.34, p < 0.001), judgment (H = 58.23, p < 0.001), spatial cognition (H = 51.04, p < 0.001), calculation (H = 56.82, p < 0.001), and language function (H = 58.23, p < 0.001), suggesting that all the cognitive domains differed across the three groups.

**TABLE 2:** Test results for the bottom five items of the VR-E.

Domains	HC	MCI	AD	H-value	p-value	Multiple comparisons	
						HC vs. MCI	MCI vs. AD
memory	89.8 ± 9.4	65.3 ± 23.4	24.4 ± 23.3	69.34	<0.001	<0.001	<0.001
judgment	83.5 ± 12.6	61.6 ± 23.0	27.5 ± 22.5	58.23	<0.001	<0.001	<0.001
spatial cognition	84.5 ± 16.6	57.9 ± 27.7	25.2 ± 23.2	51.04	<0.001	<0.001	<0.001
calculation	90.0 ± 10.2	66.0 ± 25.7	30.5 ± 23.1	56.82	<0.001	<0.001	<0.001
language function	85.9 ± 16.1	66.7 ± 27.2	35.2 ± 29.1	40.21	<0.001	<0.001	<0.001

Data are presented as mean ± standard deviation.

AD, Alzheimer’s disease; HC, older healthy control; MCI, mild cognitive impairment; VR-E, Virtual Reality-Based Cognitive Function Examination.

**FIGURE 3:**
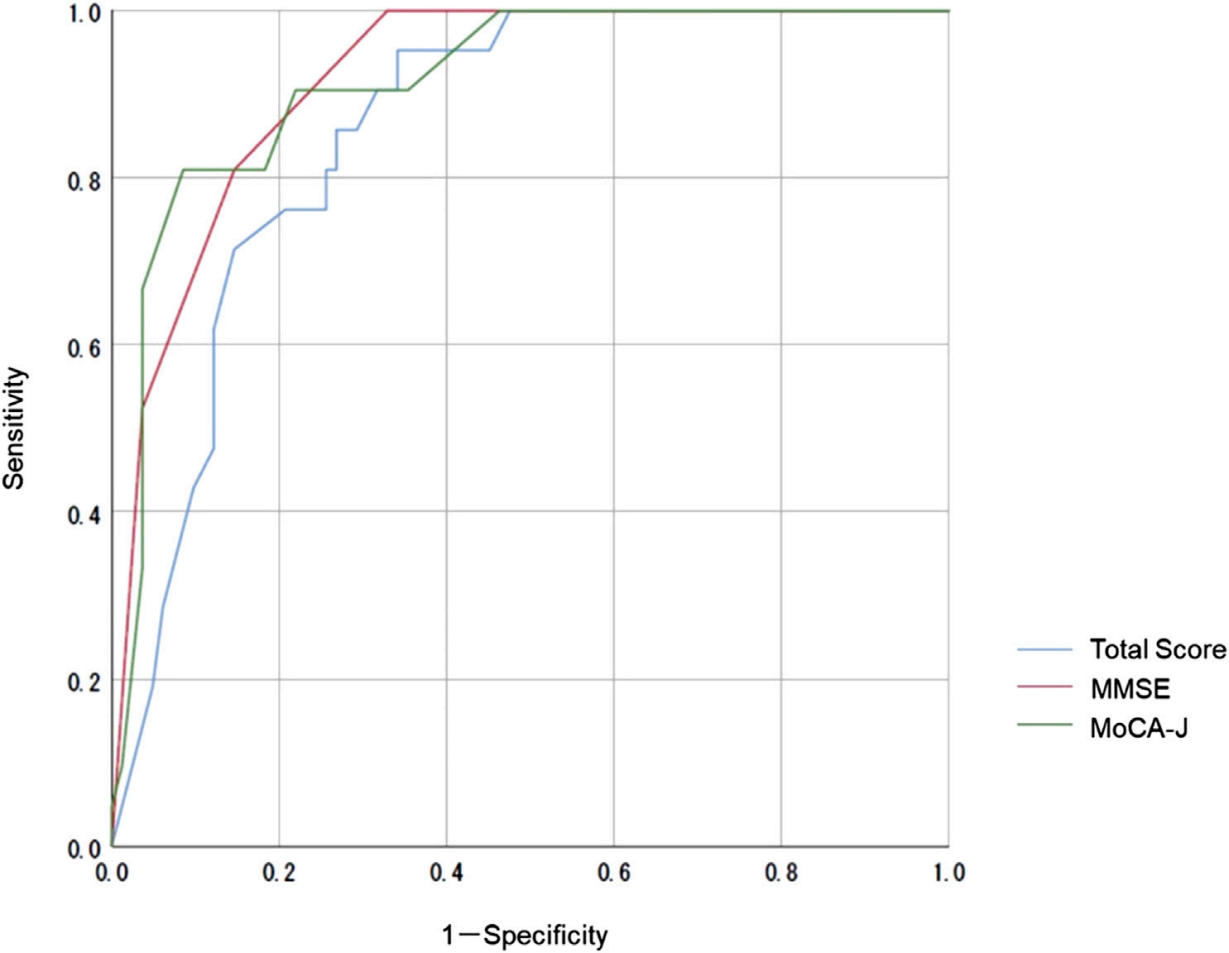
Receiver operating characteristic curves for the MoCA-J and VR-E scores of the MCI and HC groups. HC, older healthy control; MCI, mild cognitive impairment; MMSE, Mini-Mental State Examination; MoCA, Montreal Cognitive Assessment; VR-E, Virtual Reality-Based Cognitive Function Examination.

**FIGURE 4:**
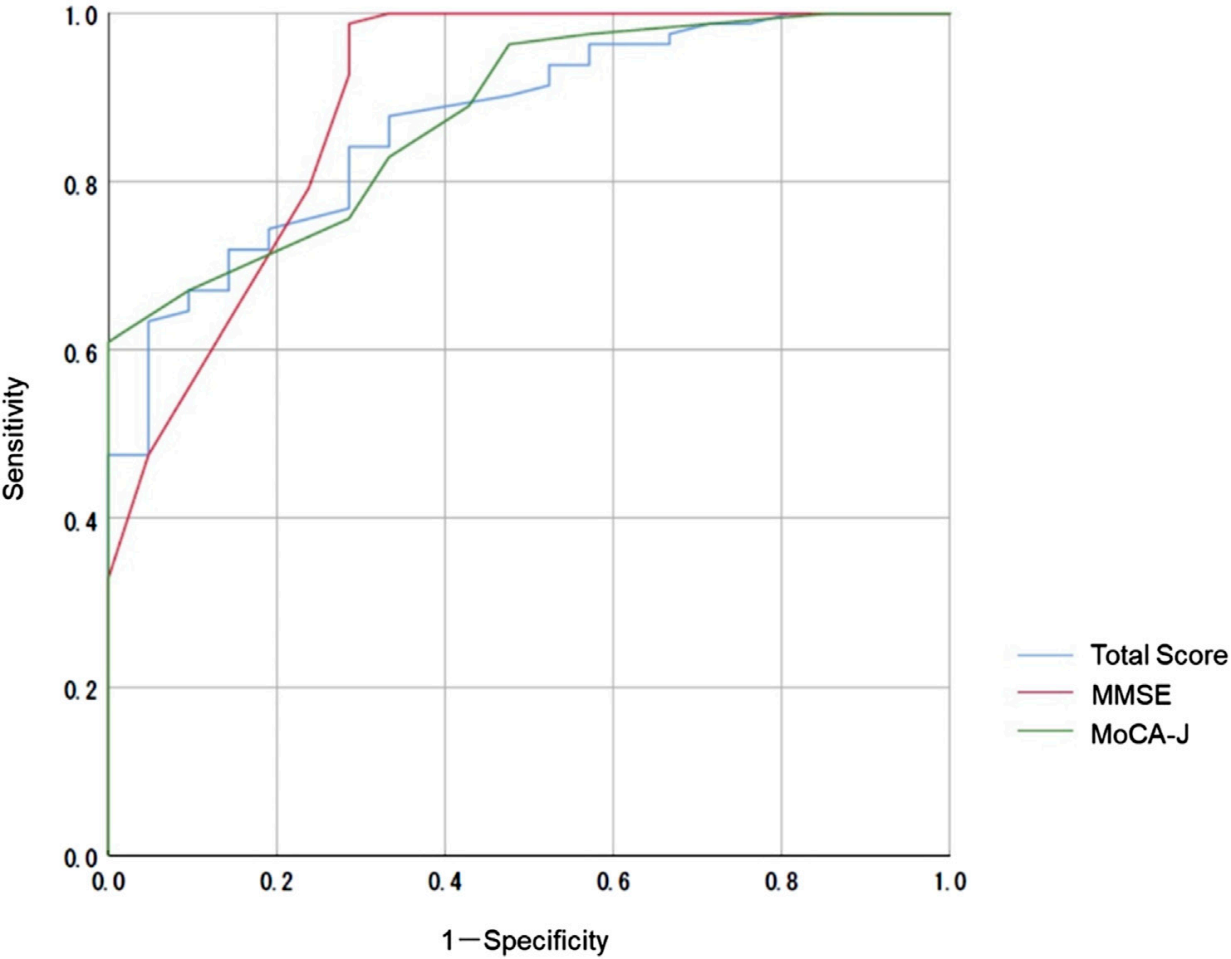
Receiver operating characteristic curves for the MoCA-J and VR-E scores of the AD and MCI groups. AD, Alzheimer’s disease; MCI, mild cognitive impairment; MMSE, Mini-Mental State Examination; MoCA, Montreal Cognitive Assessment; VR-E, Virtual Reality-Based Cognitive Function Examination.

**FIGURE 5:**
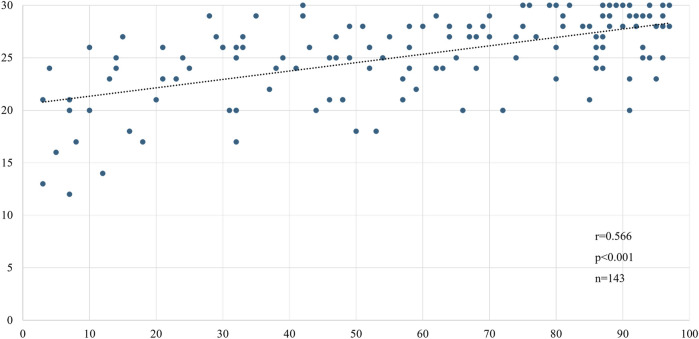
Correlation between MMSE and VR-E scores. MMSE, Mini-Mental State Examination; VR-E, Virtual Reality-Based Cognitive Function Examination.

**FIGURE 6:**
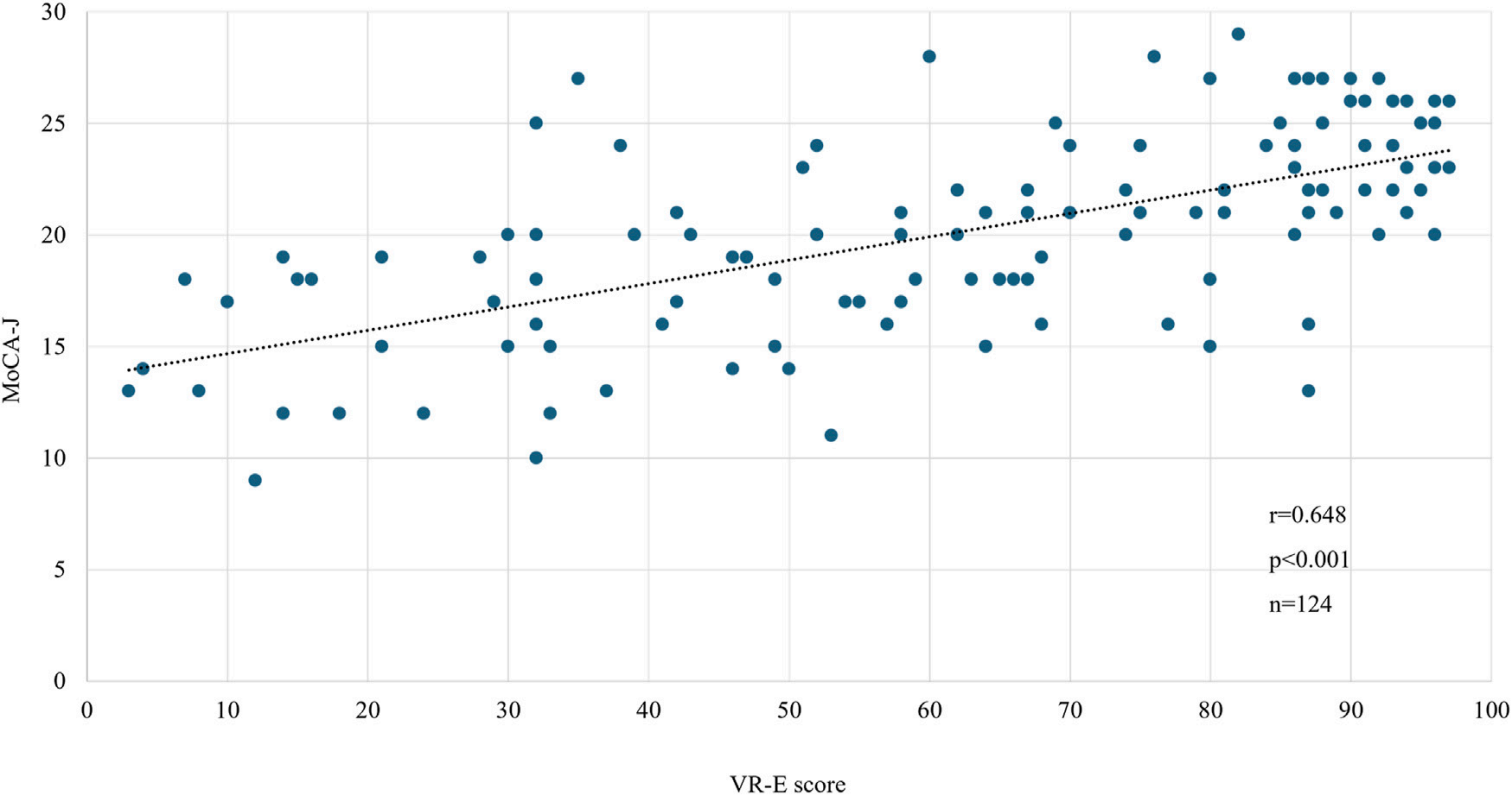
Correlation between MoCA-J and VR-E scores. MoCA, Montreal Cognitive Assessment; VR-E, Virtual Reality-Based Cognitive Function Examination.

We compared the MoCA-J results with the VR-E scores. The ROC curves for the MoCA-J and VR-E scores for the MCI and HC groups are shown in Figure 3. The AUC was 0.915 (95% CI: 0.852–0.977) for the MoCA-J scores and 0.857 (95% CI: 0.783–0.931) for the VR-E scores, indicating that the MoCA-J scores had better diagnostic accuracy than the VR-E scores. Figure 4 displays the ROC curves for the MoCA-J and VR-E scores for the AD and MCI groups. The AUC was 0.878 (95% CI: 0.807–0.949) for the MoCA-J and 0.870 (95% CI: 0.795–0.945) for the VR-E scores, indicating the VR-E scores had diagnostic accuracy equivalent to those of the MoCA-J scores. Figure 5 shows the correlation between the MMSE and the VR-E scores, and Figure 6 presents the correlation between the MoCA-J and the VR-E scores. The correlation coefficient between the MMSE and VR-E scores was 0.566 (p < 0.001), and that between the MoCA-J and VR-E scores was 0.648 (p < 0.001), which indicated a moderate correlation in both comparisons.

## 4 Discussion

This study evaluated whether a VR-E could be used to screen for MCI in individuals with HC and AD. Significant differences in the VR-E scores were indicated among the AD, MCI, and HC groups. The AUC of the VR-E scores for the HC and MCI groups was 0.86, and that for the AD and MCI groups was 0.87, suggesting that the results were as good as those of the MoCA-J. These findings suggested that the VR-E is a useful test for screening MCI. The VR-E only takes approximately 5 min to complete, and the test can be performed by a single person without an examiner. Therefore, it can be used to screen for MCI in older individuals living in the community.

Previous studies have demonstrated the potential of eye-tracking technology for assessing cognitive function. Oyama et al. (2019) reported an AUC of 0.845 (95% CI: 0.73–0.96) for cognitive scores via eye tracking for the MCI and HC groups. Tadokoro et al. (2021) reported that the AUC of the total score using eye tracking for the MCI and HC groups was 0.75 (95% CI: 0.64–0.86) and that for the MCI and AD groups was 0.78 (95% CI: 0.65–0.91), indicating that eye tracking was useful for screening patients with MCI and AD. In the present study, the AUC of the VR-E scores for the HC and MCI groups was 0.857 (95% CI: 0.783–0.931), and that for the AD and MCI groups was 0.870 (95% CI: 0.795–0.945). In this study, AUCs higher than those determined by Tadokoro et al. (2021) and almost equivalent to those of Oyama et al. (2019) were obtained. Two other studies have calculated correlation coefficients between eye-tracking scores and the MMSE. However, no correlation coefficients with the MoCA have been shown because the most important comparison for screening for MCI is with the MoCA. Our study revealed a significant correlation coefficient of 0.630 (p < 0.001) between the MoCA-J and VR-E scores, suggesting that this method is useful for screening MCI. Jiang et al. (2022) also proposed a model that combines neuropsychological testing, electroencephalography (EEG), eye tracking, and attribute data, such as age and educational history. They reported an AUC of 0.9415 (95% CI: 0.893–0.982) for the proposed model (Jiang et al., 2022). Combining eye-tracking technology with EEG and neuropsychological testing may help improve diagnostic accuracy, although, using it as a screening tool is difficult because of increased testing time and the effort required. Therefore, it is essential to screen for MCI using eye-tracking technology alone, and VR-E has the potential to be an effective MCI biomarker.

Recently, there have been reports about the early detection of dementia using artificial intelligence (AI) and machine learning. Giannouli and Kampakis (2024) demonstrated machine learning data obtained from neuropsychological tests of older adults with neurocognitive disorders could predict dementia. In particular, they reported that financial management skills are important in diagnosing dementia. However, Giannouli (2023) also determined that older adults have negative attitudes towards AI in psychological assessments. In addition, Tragantzopoulou and Giannouli (2024) reviewed the effectiveness of various neuropsychological assessments that evaluate spatial perception and navigation skills. They reported that virtual reality is important for accurately assessing cognitive decline and improving the accuracy of diagnoses for neurodegenerative diseases such as AD. This study employed a cognitive function test using a virtual reality device, and the results seem to reinforce the findings in their review. However, this study did not use technologies such as AI or machine learning. In the future, diagnostic accuracy should be further improved by considering the development of a system that combines eye tracking technology and AI that is user friendly for older adults.

The VR-E also evaluates memory, judgement, spatial awareness, calculation, and language function. Artemenko et al. (2024) reported that although basic and symbolic numerical processing ability is generally maintained in healthy older adults, the processing speed of multiple-digit numbers slows down with age, which is in line with the general decline in processing speed. In this study, we did not conduct a detailed examination of the association between age and calculation task scores. If it becomes possible to distinguish between a decline in calculation ability due to cognitive decline and a decline in calculation ability due to age, we believe that diagnostic accuracy of cognitive decline will improve even further. Moreover, this study was conducted only with Japanese subjects and lacks a cross-cultural comparison.

The study participants were patients who visited a medical institution with memory loss as their main complaint. Many patients visit a medical institution when their forgetfulness has progressed to the MCI stage; thus, more participants were in the MCI group than in the HC and AD groups. Ikejima et al. (2012) reported that dementia prevalence by age group increases with increasing age, with a prevalence of 77.7% in those aged ≥95 years. This is also why the participants were grouped unevenly during the clinical trial, which was conducted over a fixed period of 7 months. The mean ages of the participants in this study were 69.5 ± 10.3, 76.8 ± 7.7, and 84.8 ± 4.9 years in the HC, MCI, and AD groups, respectively, which is in line with the incidence and prevalence of MCI and AD increasing with age. In addition, the number of participants biased toward MCI, and differences in mean age were problematic and need to be examined in the future. Future research should increase the number of participants, and age-adjusted results should be presented.

The educational history of study target groups is also an important indicator. In this study, it was not possible to obtain the educational history of all participants. In the future, the results need to be presented with adjustments for educational history. Furthermore, participant medication histories are also an important point to consider. Although patients with AD are not administered disease-modifying drugs for dementia, many of the participants in this study were already taking anti-dementia drugs such as donepezil hydrochloride. Therefore, the effects of medication cannot be ruled out. It will be necessary to determine the medication history of all the participants in the future results presentations.

Huang et al. (2021) reviewed papers that used the CDR to diagnose MCI and dementia. They support the usefulness of the CDR for diagnosing MCI and dementia, but point out that factors such as age, educational level, prevalence of MCI and dementia, residence in developing countries, and lack of observer information may affect diagnostic accuracy. Furthermore, among the 15 papers reviewed in this study, eight were from Brazil and the United States, and only three were from Asia (Singapore, Hong Kong, and Sri Lanka). Additionally, many studies cited in Huang et al.‘s review utilized databases or community data, with limited data from healthcare facilities. The CDR relies on clinical physicians’ subjective judgements and interviews with informants rather than objective cognitive test scores, therefore does not provide sufficient evidence for accurately diagnosing MCI. In this study, we adopted a more stringent diagnostic protocol based on the widely accepted criteria proposed by Petersen et al. (1999). The aim was to distinguish individuals clinically diagnosed with MCI from healthy older adults by adopting more stringent internationally accepted criteria. In the future, we plan to investigate whether the VR-E score can distinguish participants classified as CDR 0 and CDR 0.5 in the CDR, and whether the VR-E can distinguish the severity of dementia, thereby further highlighting the usefulness of the VR-E.

MCI is situated between healthy older people and people with dementia and is underdiagnosed in the community. Therefore, MCI screening within the community is critical. In the community MCI screening conducted easily and quickly without the need for an examiner is crucial; eye tracking-based cognitive scales may be a viable solution. However, many older adults have problems with their vision, which need to be solved; for example, measurements cannot be performed in older adults with ptosis or strabismus. It is possible to test older people who wear glasses; however, it is difficult to test older people whose vision is significantly impaired. These problems need to be resolved in the future. Furthermore, the reproducibility of this device was not evaluated. Therefore, the reproducibility should be verified in future studies. This study utilized data from three medical institutions, and the MMSE and MoCA-J were administered by experienced medical professionals (nurses, speech therapists, and clinical laboratory technicians) at each institution. However, the reproducibility between facilities and between medical professionals has not been examined. Further studies are needed to examine reproducibility, such as by administering the tests multiple times.

This study verified that the VR-E was sufficient for MCI screening. The VR-E is expected to be used as a screening tool for MCI in medical facilities, local communities, community centers, and health screening centers. Further improvements in diagnostic accuracy are expected by accumulating more data on older individuals. We would like to conduct research with a system that is quicker and easier to use.

There are reports on cognitive function tests using eye-tracking technology; however, most of those studies tested visual memory. The VR-E can assess various cognitive domains, including memory, judgment, spatial cognition, calculation, and language function. The VR-E also had the same diagnostic performance as the MoCA, thus, making it potentially useful for MCI screening.

## Data Availability

The original contributions presented in the study are included in the article/supplementary material, further inquiries can be directed to the corresponding author.
